# Development of a mentorship framework guide to promote the acquisition of interprofessional education and collaborative practice competencies during undergraduate training: a mini-Delphi cross-sectional study

**DOI:** 10.21203/rs.3.rs-3923664/v1

**Published:** 2024-03-01

**Authors:** Aloysius Gonzaga Mubuuke, Faith Nawagi, Scovia Nalugo-Mbalinda, David Musoke, Rebecca Nekaka

**Affiliations:** Makerere University; Makerere University; Makerere University; Makerere University; Busitema University

**Keywords:** Interprofessional education, undergraduate students, faculty mentors, mentorship guide

## Abstract

**Background:**

The current global burden of disease which includes emerging and re-emerging diseases calls for interprofessional partnerships and team work to work collaboratively to solve community health problems. Inter-professional collaboration needs to start with training whereby learners are mentored in inter-professional teams and collaborative care. Many guidelines do exist in teaching and learning but faculty often do not have guidelines on how to mentor learners to acquire the needed inter-professional competencies. This study aimed at developing a mentorship guide for faculty to enable them to ably mentor learners in the acquisition of interprofessional competencies.

**Methods:**

It was a cross-sectional study involving both students and faculty mentors. Questionnaires were distributed to undergraduate students and the mentors at Makerere University College of Health Sciences and Busitema University Faculty of Health Sciences. Data from the participants was used alongside literature to develop the interprofessional education mentorship guide for faculty mentors. The guide was validated by a panel of experts.

**Results:**

From this study, students reported limited knowledge of the IPE core competencies and the faculty mentors corroborated this finding. Mentors did not directly give any feedback specifically targeting the IPE core competencies, though some of them unknowingly talked about some of the IPE competencies. The key challenges identified from students and faculty included limited training IPE and IPE core competencies and lack of guidelines for faculty mentors which they can follow to mentors students adequately across all the expected IPE competencies.

**Conclusion:**

There was limited mentorship in IPE competencies. Findings from this study alongside literature and expert validation, a framework guide for mentors in relation to IPE competencies has been proposed.

## Background

Inter-Professional Education (IPE) can be defined as an educational practice in which learners from two or more professions learn together; with, from, and about each other during certain aspects of their training with an aim of training health professionals to have skills in inter-professional collaborative practice [1]. Inter-Professional Education (IPE) has been defined by WHO as that occasion when students from two or more professions in health and social care learn together; with, from, and about each other during all or part of their professional training, to cultivate the idea of collaborative practice for providing quality client- or patient-centred health care [2].

This form of training prepares health professions students to gain skills in interprofessional collaborative practice[3]. Therefore, while inter-professional education (IPE) relates to learning in inter-professional groups, inter-professional collaborative practice (IPC) relates to working in inter-professional groups as a result of learning together through IPE. According to the Interprofessional Education Collaborative (IPEC), there are four core IPE competencies [4]. These include: 1) Values and Ethics, 2) Roles and responsibilities, 3) Inter-professional communication, and 4) Teams and teamwork. Values and ethics for interprofessional practice relate to working with individuals of different health professions to maintain a climate of mutual respect and shared values. Roles and responsibilities speak to the utilization of knowledge of one’s own role and those of other professions to appropriately assess and address the health care needs of patients thus advance the health of populations. Interprofessional Communication involves communication with patients, families, communities, and professionals in health and other fields. This should be done in a responsive and responsible manner that supports a team approach to promote health holistically. Teams and Teamwork involves applying relationship-building values and the principles of team dynamics to perform effectively in different team roles in given health interventions.

In addition to the IPE competencies described above, the European Interprofessional Education Network (EIPEN) has further developed five key competencies for Interprofessional Collaboration (IPC). These IPC competencies focus on the care of individual clients and their networks, client groups, and population-wide interventions [5]. Key competencies pertain to key areas of practice: consult and collaborate, plan and manage, handle issues and opportunities, refer and transfer, reflect and evaluate [5]. IPC involves communication and decision-making to enable a joint effort of influence on knowledge and skills on the desired outcome [6]. The various elements of IPC include; responsibility, accountability, coordination, communication, cooperation, assertiveness, autonomy, and mutual trust and respect [7]. These combined lead to working in Interprofessional teams, which is an important factor for the improved quality of health care and patient outcomes [1].

Team effectiveness is attributed to various factors such as open communication, blended professional cultures, autonomy, equality of resources coupled with one’s perception about the importance of their role to the team [8]. However, it is crucial to note that poor interprofessional collaboration can negatively harm the quality of patient care [9]. Therefore, it is key to acquire skills to work in interprofessional teams through IPE for quality health care.

Despite the benefits of IPE/IPC, health care professionals globally, even in Africa, have often been training in silos with minimal emphasis on IPE [10]. A few institutions in Africa have made strides in the integration of IPE competencies in the curriculum through didactic and experiential learning, especially in community-based field attachment modules and within the training of the basic sciences [11]. However, efforts to offer IPE and build frameworks that include mentorship to guide the acquisition of IPE/IPC competencies among learners especially in Africa remain minimal, yet key [12]. Mentorship requires faculty to guide learners through structured means as a way of inculcating IPE competencies. In Africa, efforts have been put in place to have some form of learning and teaching IPE competencies with variable success [13]. However, in many learning opportunities where students from different professions are put together, learning still occurs in silos [14]. Students from different health professions may sit together in class but still learn independently with hardly any opportunities to learn IPE competencies [15]. This significantly denies students an opportunity to learn and acquire IPE and IPC competencies that they might need to provide quality health care after their training. Nevertheless, having students from different health professions getting placed in the same class doesn’t necessarily translate into inter-professional education. There has to be a structured way that promotes inter-professional learning in teams to occur [12]. Focused mentorship by faculty for acquisition of IPE competencies can be one way of addressing this. The Africa Interprofessional Education Network (AFRIPEN) has recently been formed to foster IPE/IPC in health workforce training and effective health systems in Africa but lacks frameworks to guide IPE/IPC learning in various domains [16]. Faculty play a crucial role in guiding students.

Like in many parts of the world, the training of health professionals in Africa has largely remained in silos whereby each professional discipline accounts for its learners and they learn separately from other disciplines. Even in situations where attempts have been made to foster IPE, it has been simply putting students from the different professions together in the same lecture room or community site without deliberate efforts to ensure that they acquire IPE core competencies. There is limited mentorship in IPE competency acquisition during undergraduate training in many African institutions. Although mentorship in Africa is one of the key academic activities in various health professional training institutions, this has also largely remained within professional silos with minimum attention given to mentorship specifically targeting the acquisition of IPE competencies. In this study, we aim to address this gap by developing a mentorship framework guide for mentors in health professional training institutions that will specifically target the acquisition of IPE competencies.

### Theoretical Framework

The study was underpinned by the social constructivism theory and the Zone of Proximal Development. These were used because, social constructivism emphasizes individuals working together to construct and develop ideas through dialogue, building on prior knowledge and understandings. [17] Here, the importance of culture, context, and social interaction are key. For the social constructivist, reality cannot be discovered: it does not exist before its social invention. Knowledge is socially and culturally constructed [17]. Individuals create meaning through their interactions with each other and with the environment they live in. Learning is a social process. It does not take place only within an individual, nor is it a passive development of behaviors that are shaped by external forces [19]. The social constructivism theory by Vygotsky also goes ahead to emphasize the Zone of Proximal Development. It postulates that an individual will have skills/abilities they develop on their own but cannot perform them independently because they will need guidance from someone who has mastered the skill already to enable them to learn and be able to practice independently [20]. Therefore, meaningful learning occurs when individuals are guided by experts in a given field, engaged in social and collaborative activities, and optimally if done interprofessionally [17].

## Methods

### Study design

It was a cross-sectional study conducted at Makerere University College of Health Sciences and Busitema University Faculty of Health Sciences. Makerere University is the eldest health professions education training institution in Uganda located in the capital city, Kampala while Busitema University is a rural based university with a relatively younger health sciences faculty. The study involved both undergraduate students and the faculty mentors.

### Study setting

The study was conducted at two health training institutions in Uganda including: Makerere University College of Health Sciences and Busitema University Faculty of Health Sciences. Makerere University College of Health Sciences is the oldest institution that trains health professionals in Uganda and the entire East African region and it comprises of five schools namely: School of Medicine, School of Public Health, School of Health Sciences, School of Biomedical Sciences and School of Dentistry. From among these schools, there are various undergraduate and postgraduate programmes that span different professions. Busitema University Faculty of Health Sciences is a relatively young rural based institution that also trains a number of different health professions including medicine, nursing and anaesthetic officers.

### Participants

The participants included undergraduate health sciences students and faculty from the two institutions who were invited to complete an online survey through their e-mail address which had the link to the survey. All undergraduate students regardless of health sciences discipline being pursued were eligible to participate in the study and all faculty from the two institutions were also eligible to participate in the study.

### Data Collection

The distributed questionnaire had both close-ended and open-ended responses, hence the data collected was both quantitative and qualitative. Two questionnaires were developed, one for students and the other for the faculty. The data collection tools were purely online and self-administered. The tools were first piloted with a few students and faculty before distributing them to the participants via their e-mails. All students and faculty at the two participating institutions have institutionalized e-mail addresses through which the tools were disseminated.

### Data Analysis

For the qualitative responses to the open-ended questions, inductive thematic analysis was used to analyze the data where common emerging codes were related to each other to generate themes. This was done with assistance from Atlas software. For quantitative data, SPSS statistical package was used for analysis. From the raw data, frequencies, means and SDs were determined.

### Development of the mentorship framework guide

Information from the quantitative and qualitative responses from participants alongside the reviewed literature were utilized to develop the IPE mentorship guide to facilitate the acquisition of interprofessional education competencies that is feasible in low-resource settings. Using a modified Delphi technique, the developed guide was then presented to a panel of five experts in IPE and health professions education for further validation and reach a consensus before finally coming up with the eventual IPE mentorship guide that has been presented in this study. Delphi technique refers to a process used to arrive at a group of opinions or decisions by surveying a panel of experts through a series of various questions.

### Ethical Considerations

Ethical approval to conduct was granted by the Mulago Hospital Research Ethics Committee (MHREC-2023–100) and final approval was granted by the Uganda National Council for Science and Technology (HS2920ES). Informed consent was obtained from all participants prior to beginning the survey and there was no identifying information on the online tools. Before proceeding to complete the online tool, each participant had to first read the consent and agree to participate.

## Results

The purpose of this study was to explore experiences of students and faculty regarding mentorship in IPE/IPC competencies and utilize these experiences to develop a guide for mentors to facilitate the acquisition of IPE/IPC competencies among undergraduate health sciences students. The key findings from the study are presented below:

### Results from the Students` Survey

A total of 248 undergraduate students responded to the survey. The key socio-demographic characteristics of the students across the two institutions (Makerere and Busitema) are summarized in Table 1 below:

The mean age of the students was 24.7 years (SD=5.1) and majority were from Year three of study (SD=0.9). From the table above, it can be observed that majority of the students were pursuing medicine when compared to other health sciences disciplines, but the table also demonstrates that there was a fair representation of students from a variety of health sciences disciplines, underpinning the interprofessional nature of the survey. Majority of the students from across all health disciplines had no prior IPE training with a mean age of 24 years. However, it should be noted that some of the students had some knowledge of IPE (40.3%, n =100)

### Responses from the students regarding mentorship in Interprofessional Education Competencies

More than half (59.7%, n=148) of the students reported to have no mentor while more than about a quarter (40.3%, n=100) reported to have had a mentor at one time. The frequency of the students meeting their mentors was mainly occasional characterized by statements such as sometimes, rarely, once a semester, and irregular. For the students who had had mentors at some point in time, they mentioned that they would meet their mentors occasionally as individuals but not with students from other disciplines. Analysis of the qualitative responses from students resulted into three key themes namely:

### Theme 1: Topics discussed with mentors

The responses indicated that when some of the students occasionally meet with their mentors, the discussion areas are centred around academics and career. Interpersonal skills, research and leadership. The following responses represent this observation.

We mainly discuss issues to do with academics and how they stress us and how to handle it. Occasionally, we discuss with my mentor how to go through challenges at medical school and excel

- Busitema University Student

I have discussed a lot with my mentor’s issues to do with excelling in my academics, conducting good research, data analysis, social life, work-life balance and how to become a good leader in medical school

- Makerere University Student

From the above responses which represent what swept through most student responses, it appears like mentors put much emphasis on academic related skills. Though some effort is put on other skills like leadership and social life, one can hardly see any effort put towards specifically the acquisition of IPE competencies among the students.

### Theme 2: Gaining IPE skills during mentorship relationships

Having explained to them what IPE was about, the students were asked about how they thought their mentors can help them gain IPE skills. Many of them pointed to the use of simulation based learning, group discussions, journal clubs on IPE, joint clinical ward rounds involving students from different health disciplines, role modelling from mentors of the different professions. The following responses reflect some of these observations:

Since IPE skills seem to be aimed at fostering collaboration between different health professions, it would be good to see from our mentors how they collaborate in form of role modelling

- Makerere University Student

We have simulation labs and it would be good to first show us the application of some of those IPE competencies before we go to the wards, for example our mentors can identify a case involving different health professionals caring for a patient and show us the roles that each brings to the table

- Busitema University Student

I think since IPE aims at making sure that we work together as health workers, let our mentors organize some joint ward rounds where for example medicine students clerk a patient together with nursing and pharmacy students…. there you can see how each of us can bring their knowledge to care for the patient, but in most cases we learn in our own disciplines

- Makerere University Student

From the student responses above, one can see a lack of learning opportunities for students to acquire IPE skills and some suggestions to improve this are thus suggested. The students were also asked about the IPE skills they wanted to gain during these suggested learning opportunities. The majority of the responses pointed to the following skills: communication skills, leadership, working together and collaboration with other professions. Although not comprehensive enough, the students pointed to some of the key aspects of the core IPE/IPC competencies.

### Theme 3: Improving the acquisition of IPE competencies

The last theme that emerged out of the student responses related to the ways in which the learning of IPE skills can be improved. From the responses, assessment of the IPE skills besides the academic competency as well as having some guidelines for mentors that relate to IPE were recurrent as evidenced in the responses below:

The issue of IPE seems to be new to not only us but also may be our mentors. My mentor never used to talk about IPE the few times I met her and perhaps both students and mentors need some form of guidance or guidelines which mentors can follow to give us feedback on IPE skills since most focus on academic excellence.

- Makerere University Student

There should be deliberate efforts by our mentors to teach us IPE skills as they seem to be very important, but we are never taught these things….and as long as you are not taught, you will not be assessed even. Therefore, the mentors can at least try to assess some of these during the semester or during OSCEs to see if we have acquired them, but as long students are not assessed, they may not learn them.

- Busitema University Student

Since we are meant to be meeting with our mentors frequently, they can always give us feedback on how we fair with these IPE skills. For example, if I am on the ward clerking a patient, the mentor can ask me how I interacted with the patient and other professionals that were seeing that patient and how we arrived at a diagnosis.

- Makerere University Student

From the responses above, one can see that improving the acquisition of IPE competencies needs mentors to perhaps give feedback that specifically targets the IPE competencies.

### Results from the Faculty Survey

In order to triangulate our findings regarding mentorship in IPE skills, we also sent out a survey to the faculty mentors. The demographics of the faculty mentors are shown in Table 2 below:

From the table above, it can be observed that majority of the faculty had no prior training in IPE at 84.6% (n=44) while only 15.4% (n=8) had had prior training in IPE. The faculty mentors who responded also reflected a range of professional disciplines just as it was reflected from the students in this study.

### Responses from mentors regarding IPE competencies

More than half of the faculty mentors (65.4%, n=34) reported to have had a mentor while about a quarter (34.6%, n=18) reported to have had no mentor. The frequency of meetings between mentors and mentees was very variable ranging from never meeting at all to perhaps meeting only once in a semester. The qualitative responses from faculty mentors generated two major themes:

### Theme 1: Challenges with mentorship in IPE competencies

A key theme that emerged related to challenges with mentorship that focuses on the acquisition of IPE competencies. From the faculty responses, some key challenges were identified which resonated throughout most of the responses. These included: limited time to meet mentees, limited knowledge and skills to mentor students, inadequate knowledge on what IPE competencies are and lack of some guidelines to enable faculty mentor students to acquire IPE skills. The following responses illustrate some of these challenges:

The truth is that those times I meet my students assigned to me to mentor, we talk about their academic progress and challenges they are meeting with their learning…. I think this is what am supposed to do as a mentor, but the idea of mentorship in IPE is a new concept to me. I am not sure if by meeting my student and talking about this, it is part of the whole thing of IPE

- Makerere University Faculty Mentor

I have students who approach me for mentorship and others are assigned, but they just come with no guidelines at all for us as mentors. I do not know if am doing the right thing and nobody has talked to me about this IPE issue….it would be good may be to train us as mentors and tell us exactly what we should focus on to ensure that students acquire the IPE skills being talked about

- Busitema University Faculty Mentor

I have heard of IPE in many meetings and it seems it is a very important concept and we are required to mentor students to acquire IPE skills to achieve better patient outcomes. However, we are just expected to mentor students without any guidelines on how to do it and we may also need training in IPE

- Makerere University Faculty Mentor

From these responses, it can be seen that like the students pointed out, the faculty mentors also agreed that there needs to be some kind of training in IPE as well as guidelines that specifically relate to IPE competencies.

### Theme 2: Improving IPE mentorship among students

Another theme that emerged was about improvement of mentorship in relation to the acquisition of IPE skills among undergraduate students. Key suggestions were evident in the responses, but most of them rotated around training faculty mentors in IPE and what IPE competencies are as well as coming up with some guidelines to help the mentors to focus on the targeted IPE competencies. The following responses reflected these observations:

As faculty mentors, we need some preparation before students are sent to us, some kind of orientation into what I should focus on. Specifically, for IPE, it is a good thing but as faculty we need training in IPE to know what it is and what competencies are in there so as to enable us mentor the students well…. otherwise if we do not know, the students will not gain these skills yet they seem to be very useful

- Makerere University Faculty Mentor

Mentors should be on the same page. If we want to promote IPE as part of student mentorship, can we define what specific skills in IPE that we want then we train all faculty to focus on these skills if we want to avoid being everywhere. Therefore, some form of guidelines showing us what to focus on that relates to IPE will make our lives better as we mentor our students to practice this interprofessional practice that we are advocating for

- Busitema University Faculty Mentor

What resonates from the above responses is the need to train mentors in IPE and perhaps have some form of guidelines for them that specifically relate to IPE competencies.

### The IPE Faculty Mentorship Framework Guide

The IPE mentorship framework guide is shown in Table 3 below. This guide resulted from the responses of the students and faculty as well as a rapid assessment of literature on IPE competencies and mentorship in relation to these IPE competencies with a final validation by a panel of experts. A modified mini-Delphi technique was employed in which the original framework guide formulated was sent to five experts in IPE and requested to remove/add any descriptor items from the framework in relation to each IPE core competency domain. Two rounds were conducted to reach some acceptable level of consensus. The framework guide presented in Table 3 is as a result of this final step.

## Discussion

The purpose of the present study was to explore student (mentee) and faculty (mentor) experiences of mentorship in relation to IPE competencies, and to utilise these experiences to develop a framework guide to enhance the acquisition of IPE competencies during mentorship relationships. The developed framework was further validated by a mini-Delphi technique using IPE experts. Responses from students demonstrated that majority had not received any formal training in IPE and do not receive feedback from their mentors about IPE competencies. Although some students had some knowledge about IPE, they could not clearly articulate the core IPE competencies. The students’ responses about limited knowledge on IPE competencies and the fact that mentors do not focus on IPE competencies corroborated with responses from faculty mentors. The faculty also reported limited knowledge on IPE and IPE core competencies which hinders their ability to mentor students. This can be explained by the fact that there is no formal training of faculty in IPE despite the fact that the training institutions are emphasizing IPE during training as a way of promoting future collaborative practice. In many other places, a similar observation has been reported that limited training in IPE hinders the effective learning of IPE related outcomes [6, 18]

Knowledge of the IPE core competencies is crucial and if students are to acquire IPE competencies through their mentors, then mentors need to be trained on what these competencies entail as has been reported by other scholars [10]. The limited training and hence limited knowledge of IPE competencies on the side of faculty mentors could perhaps offer an explanation as to why students reported receiving limited mentorship in IPE competencies. IPE has been reported to now be an important component of training health professionals [20]. The core IPE competencies as suggested by WHO play a crucial role in nurturing health professionals that will be able to work in interprofessional teams to deliver high quality patient-centred care [2, 5]. One way of imparting these IPE core skills is through effective mentorship relationships in which mentors follow up mentees targeting their learning of IPE competencies. However, in the absence of training mentors in IPE and the related core competencies, it becomes a challenge for the mentors to shape the students. It should be noted however that some of the mentors actually may have talked about some of the IPE competencies though unknowingly. Aspects such as respect and communication are part of the IPE competencies. However, there is need to deliberately train faculty mentors and make them aware of the all the key IPE core competencies and give them guidance on what to consider when imparting IPE skills among their students.

Mentorship interactions do not occur in a vacuum, but are rather situated within a community of learning. This community of learning may have various interacting factors that can influence student growth in relation to the acquisition of IPE competencies. The fact that students in this study as well as faculty mentors pointed out the need to have guidelines for mentorship with a special focus on IPE competencies should not be ignored. This observation has been alluded to from elsewhere [20, 21]. The need to have guidelines for mentorship that target IPE core competencies is thus urgent. Such guidelines could emphasise the core IPE competency domains along with key descriptors that explain each IPE competency. By following these descriptors, mentors will be able to give students feedback that targets acquisition of each IPE competency.

In this study, having guidelines for feedback delivery during mentorship interactions was also proposed by the mentors themselves. From this study, a framework guide has thus been proposed to assist the mentors to construct feedback to their mentees that targets each IPE core competency. This guide is aimed at acting as a mediating tool for mentors during mentorship relationships, and at ensuring that mentors frame feedback that specifically targets the acquisition of IPE/IPC competencies among students. The strength of the guide lies in its structured nature that allows mentors to specifically focus on certain areas against each IPE competency domain. Structuring the guide is likely to achieve two things: (i) it may be easily acceptable to faculty, and feasible to implement; and (ii) it could be an avenue through which mentees receive feedback across all targeted IPE competencies as mentors shall be following this guide during mentorship. This framework guide can potentially enhance the mentorship relationships and facilitate the acquisition of key IPE core competencies. Overall, this study supplements literature on mentorship specifically focusing on the acquisition of IPE competencies. The fact that the study has pointed out the need for training faculty mentors in IPE gives pointers to institutions on where to start from if they are to implement successful IPE mentorship programmes. The guide developed from this study provides a building block for faculty mentors that can help them focus their mentorship activities in relation to the core IPE competencies.

### Study limitations

This study was conducted in two institutions, but social and academic contexts may differ across different institutions, and therefore the findings may not be generalizable, a major limitation of the study. In addition, the model/ framework developed did not consider other areas of student support such as peer mentorship/feedback that could be vital in promoting IPE competencies.

### Further research

The implementation of the IPE mentorship guide developed from this study and the evaluation of its feasibility and potential impact on the outcomes of acquisition of IPE competencies among students were beyond this particular study, but are recommended moving forward.

## Conclusions

The present study explored student and faculty experiences of mentorship in relation to learning IPE competencies. Students generally reported limited mentorship in the IPE competencies, and faculty pointed to a lack of adequate knowledge in IPE as well as absence of guidelines for mentors that focus on the various IPE competencies. A framework guide that specifically focuses on key IPE competencies has thus been developed from the study to facilitate the work of mentors when guiding students to obtain the targeted IPE core competencies.

## Figures and Tables

**Table 1:** Student characteristics, N=248

Variable	Frequency (n)	Percentage ( %)
**Institution**		
Makerere University	80	32.3
Busitema University	168	67.7
**Gender**		
Female	112	45.2
Male	136	54.8
**Program of Study**		
Medicine	151	60.9
Nursing	50	20.2
Anesthesia	10	4
Pharmacy	8	3.2
Dentistry	7	2.8
Radiography	7	2.8
Environmental Science	6	2.4
Speech and language therapy	3	1.2
Cytotechnology	2	0.8
Optometry	1	0.4
**Prior IPE Training**		
Yes	100	40.3
No	148	59.7

**Table 2:** Faculty characteristics N=52

Variable	Frequency (n)	Percentage ( %)
**Institution**		
Makerere University	45	85.6
Busitema University	7	13.4
**Level of Education**		
PhD	32	61.5
Masters	20	38.5
**Profession Discipline**		
Medicine	29	55.8
Nursing	12	23.1
Bio Medical Sciences	4	7.7
Pharmacy	1	1.9
Public health	3	5.8
Dentistry	2	3.8
Optometry	1	1.9
Others	3	5.8
**Prior IPE Training**		
Yes	8	15.4
No	44	84.6

**Table 3:** The IPE Mentorship Guide

IPE Competency Domain	Description
**Values and Ethics**	**Mentor should discuss with mentee:** **Issues of mutual respect** **Respect for other professions** **Cultural uniqueness and diversity** **Dignity for colleagues and patients** **Patient confidentiality and privacy** **Good ethical and professional conduct**
**Roles and Responsibilities**	**Mentor should discuss with mentee:** **Use of onès knowledge to deliver quality healthcare** **Understanding and performing onès role in the healthcare team** **Understanding roles of other professionals in the healthcare team** **Integrating onès unique role with roles of others in the team to deliver quality healthcare**
**Teams and Team work**	**Mentor should discuss with mentee:** Building relationships with other professional colleagues in the team Forming highly effective teams Networking with other colleagues in the team and beyond Understanding values and principles of team dynamics Maintenance of healthcare team cohesion Assigning roles and responsibilities for the good of the patient Planning and delivering patient centered care in an interprofessional team
**Interprofessional Communication**	**Mentor should discuss with mentee:** Importance of good communication skills (verbal & non-verbal) with both professional colleagues and patients Good listening skills Interpersonal communication Resolving disagreements Articulating ideas and opinions regarding best care for the patient to other colleagues

## Data Availability

The datasets used and/or analysed during the current study are available from the corresponding author on reasonable request.
